# Probiotics at the Frontline: Redefining Therapeutic Possibilities

**DOI:** 10.3390/nu18010048

**Published:** 2025-12-23

**Authors:** George Stavrou, Alexandra-Eleftheria Menni, Katerina Kotzampassi

**Affiliations:** 1 Department of Surgery, 417 NIMTS (Army Share Fund Hospital), 11521 Athens, Greece; stavgd@gmail.com; 2 Department of Surgery, Aristotle University of Thessaloniki, 54636 Thessaloniki, Greece; alexmenn@auth.gr

Recently, advances in microbiome research have emphasized the fundamental role of probiotics—in addition to their inanimate form, postbiotics, and psychobiotics, a rapidly expanding group of probiotics with psychotropic potential [1,2,3]—in restoring disturbed microbial diversity in the human body [4,5,6,7,8,9,10,11]. Much progress has been made through experimental studies in cell cultures or animal models [12,13], enabling the investigation of underlying mechanisms in controlled biological settings [14] beyond the gastrointestinal tract and the maintenance of the intestinal barrier; these setting include renal and respiratory physiology and function [15,16], bone and dental integrity [17,18], immune enhancement [10], and even modulation of psychiatric and neurobehavioral disorders [19]. This expansion of focus beyond the gut reflects a transformative shift in our understanding of host–microbe interactions and opens promising paths for future therapeutic strategies.

In this context, the current Special Issue, “Effects of Probiotics, Prebiotics, and Postbiotics on Human Health (2nd Edition),” brings together a collection of twelve contributions that cooperatively advance our understanding of these rapidly evolving topics. The Special Issue includes seven experimental studies that offer mechanistic or exploratory insights, two original articles—randomized clinical trials—focusing on psychobiotics and their impact on depression and pain, and three reviews that synthesize existing evidence; all of these will be summarized below, illustrating both the depth and the extent of current research in microbiome-directed interventions.

Vera et al. [20] highlighted the immunomodulatory properties of *Lactiplantibacillus plantarum* MPL 16 and CRL 1506 strains, enabling protection against opportunistic gastrointestinal tract infections. Using malnourished mice subjected to gentamicin treatment and then challenged orally with *E. faecalis*, a 7-day renourishment with a balanced conventional diet plus the *L. plantarum* strains was found to accelerate immune recovery and improve resistance to infection by modulating systemic and gut cytokine profiles in relation to only diet-receiving mice. Furthermore, in an in vitro model of airway inflammation, lung carcinoma epithelial cells (A549) and normal bronchial epithelial cells (16HBE), after becoming inflamed by IL-1β stimulation, were treated with viable or heat-treated *Ligilactobacillus salivarius* (LS01 DSM 22775) and *Bifidobacterium breve* (B632 DSM 24706). Both live probiotic strains—or as postbiotics—demonstrate positive results by significantly decreasing pulmonary inflammation through the inhibition of NF-κB and COX-2 pathways. The authors [21] suggest their potential for targeting airway inflammation directly by means of inhalation in respiratory diseases. Regarding healthy individuals, a probiotic regime of *Lactiplantibacillus plantarum* PBS067 (DSM24937), *Lactobacillus acidophilus* PBS066 [DSM24936], and *Bifidobacterium animalis* subsp. *lactis* BL050 [DSM 25566] or an identical placebo given as a preventive measure for cold symptom relief and immune response enhancement for a 12-week period, from December through April, was found to effectively alleviate cold symptoms [fever and muscle pain] and reduce pro-inflammatory cytokine levels [22]. Additionally, a systematic review [23] further confirmed that inactivated probiotics, from the *Lactobacillaceae* and *Bifidobacteriaceae* families exerted multifaceted anti-inflammatory effects by means of modulating cytokines expression, thus influencing immune cell signaling pathways and strengthening the epithelial barrier integrity. The authors foresee the potential of selected postbiotics strains in treating inflammatory bowel diseases, autoimmune diseases, and metabolic syndrome; however, further experimental research and long-term clinical trials, as well as thorough analyses of the synergistic effects of different strains is a prerequisite.

Regarding manipulation of microbiota in metabolic and gastrointestinal disorders, Zhong et al. [24] evaluated the efficacy of *Lactiplantibacillus* strains LTJ1 and LTJ48 in both HepG2 cells in degrading purine/nucleoside and in a hyperuricemia mouse model in order to lower uric acid levels and restore microbial balance. Probiotic strains isolated from the fermented grains of soy sauce-flavored Baijiu distillate—a traditional Chinese grain—were found to degrade > 97% of purines/nucleosides in vitro and to directly reduce uric acid production in vivo, along with restoring gut microbiota dysbiosis. This evidence positions microbiome-based modulation as an innovative prophylactic strategy for chronic hyperuricemia management and its associated comorbidities. In a loperamide-induced mouse model of functional constipation, the probiotic *Weizmannia coagulans* BC99 [formerly *Bacillus coagulans* BC99] was found to prevent functional constipation by increasing fecal water content, gastrointestinal transit rate, microbial metabolic activity, and butyric acid production, in addition to increasing gastrointestinal regulatory peptides, including motilin and somatostatin and the firmicutes-to-bacteroidetes ratio [25]. On the other hand, it was found that the detrimental role of secondary bile acids in the colon—linked to Westernized dietary patterns—could be ameliorated by means of the *Pleurotus eryngii* mushroom [strain LGAM 216] fermentation supernatant. This supernatant, when applied to the Caco-2 human colon adenocarcinoma cell line, along with sodium deoxycholate or co-cultured with colonic mucosa biopsies obtained from healthy individuals, revealed a remarkable ability to preserve the integrity of tight junctions, while modulating paracellular and transcellular permeability during exposure to secondary bile acids. Beyond this, it demonstrated a downregulation of pro-inflammatory mediators and a restoration of immune equilibrium [26].

Later, in the new era in advanced treatments, Park et al. [27] tried to elucidate the effect of the postbiotics *Lactococcus lactis* HY449 and *Streptococcus thermophilus* HY9012 on macrophage polarization and osteoclast differentiation in the human monocytic leukemia cell line [THP-1] and to assess their therapeutic efficacy in a ligature-induced periodontitis mice model. Both postbiotics reveal their properties in modulating osteoclast differentiation and macrophage polarization in a periodontitis model and were found to open a new potential therapeutic pathway, adjuvant to conventional treatment strategies. On the other hand, Kuang et al. [28] questioned whether probiotics can prevent and treat osteoporosis; in a male rat model of deoxycorticosterone acetate salt-induced osteoporosis, they found that live *Lacticaseibacillus rhamnosus* AC1 aggravated bone loss, the effect being associated with alterations in gut microbiota and disruption of the coupling process in bone remodeling. Thus, they suggest a cautious assessment of the hormonal and metabolic profile of individuals when receiving probiotic therapies for bone and mineral disorders.

Finally, regarding probiotics being neuroactive, Śliwka et al. [29], in a systematic review, analyzed the effects of psychobiotics on mental health outcomes. Although there was an overall heterogeneity in the studies they analyzed, regarding strain specificity, dosage, duration, and psychological assessment tools used, they reported significant improvements in depressive symptoms, anxiety, and stress through modulation of neurotransmitters, regulation of the HPA axis, production of SCFA, immune modulation, and rebalancing of gut microbiota. In the same manner, Menni et al. [30] conducted a more sophisticated analysis of existing literature on depression treatment with psychobiotics alone or as an add-on to antidepressants, the strict inclusion criteria being (i) depression as the primary diagnosis using psychometric tests; (ii) depression improvement as the clear primary objective of study; (iii) randomized controlled trials only with the ingredients of the placebo treatment being precisely defined. They conclude that, in particular, the multi-strain preparations and certain well-characterized single strains seem to be noticeably beneficial in alleviating depressive symptoms in adults, either alone or in conjunction with antidepressants. Finally, Tzikos et al. [31] suggest psychobiotics as a promising non-opioid add-on for comprehensive cancer pain management. In a post hoc analysis of data from a randomized, placebo-controlled trial, which originally aimed to assess the psychotropic effects of a four-strain psychobiotic regime in postoperative gastrointestinal cancer patients receiving chemotherapy, they assessed the changes in pain perception among non-depressed and depressed participants, who received either psychobiotics or placebo, along with standard analgesic regimes. They conclude that adjunctive psychobiotic therapy beneficially affects pain perception, with the most pronounced effects observed in the non-depressed individuals. These findings suggest psychobiotics as a promising non-opioid add-on for comprehensive cancer pain management and support further investigation in larger pain-targeted trials.

Collectively, the contributions of this Special Issue reflect the remarkable conceptual and translational expansion of probiotic research. At the same time, the findings highlight the necessity of rigorous strain-level characterization, appropriate dosing strategies, and treatment duration, along with careful consideration of the metabolic, hormonal, and immunological status of the host. Taken together, these contributions confirm that probiotics are at the forefront of innovative, microbiome-targeted therapies, with their diverse biological actions and expanding therapeutic potential reflecting a rapidly evolving field with profound implications for personalized therapies as well as preventive medicine.

## Data Availability

Not applicable.
